# Autism and ADHD: A Literature Review Regarding Their Impacts on Parental Divorce

**DOI:** 10.3390/children10030438

**Published:** 2023-02-23

**Authors:** Smeralda Diandra Anchesi, Francesco Corallo, Marcella Di Cara, Angelo Quartarone, Rino Catalioto, Francesca Cucinotta, Davide Cardile

**Affiliations:** IRCCS—Centro Neurolesi Bonino-Pulejo, S.S. 113, Via Palermo, C. da Casazza, 98124 Messina, Italy

**Keywords:** children, ADHD, autism, family, divorce, separation

## Abstract

Introduction: The change in family structure as a consequence of divorce can be a traumatic event for a child that can undermine his or her emotional security. For this reason, it becomes a major health concern. Many divorce-related risk factors have been identified, including attention deficits or autism spectrum disorder (ASD) in children. The aim of this review is to evaluate if and how a diagnosis of ASD or attention deficit hyperactivity disorder (ADHD) in children is associated with an increase in divorce within families. Method: Searches were performed in two databases evaluating studies focusing on articles pertaining to the topic. A total of 20 articles were found, but only 8 were included in the study according to the criteria. Results: The results showed that divorce does not appear to be specifically related to a diagnosed pathology of the child, but rather presents itself as a risk factor in certain situations. In particular, this occurs when the coping strategies required to deal with the diagnosis are dysfunctional. However, it would appear that families in which there are children with ADHD have a greater chance of divorce than families in which there is a child with a diagnosis of ASD. It may be hypothesised that in the latter case, parents receiving a diagnosis early in the child’s life have more time to develop adaptive strategies to cope with the condition than parents with children with ADHD who mostly find themselves having to deal with their child’s behavioural problems at a school age. Moreover, ASD is a disorder more likely genetic than environment-related, so parents receive more socio-medical support, and they are less likely to blame themselves or be blamed by others.

## 1. Introduction

Within the family context, especially during early childhood, the child is able to learn the basic skills that provide the foundation for future life. Prominent among these skills is attachment, which constitutes a strong and lasting emotional bond that an infant shares with a person who is fundamental to him, usually his mother, who knows and responds to his needs [1]. Early experiences are important in shaping the personality, emotionality, and sociality of individuals. When parenting is warm, attentive or positive, and characterised in a stable manner by harmony and manifested mutual appreciation, it is associated with a better level of resilience in an adverse context [2], and with a secure attachment [3]. This term refers to the readiness with which an infant uses the primary caregiver to derive a sense of security in experiencing and exploring the world.

Family shapes the personality characteristics of the child in all its parts. Family cohesion has been proven to be a key factor in child development. A study by Hammes et al. showed that children from cohesive families exhibit greater social skills, while those from conflict families display externalising behaviour such as irritability and aggression [4]. Herke et al., in one of their recent studies, found that a better relationship with family members and strong family cohesion is associated with a more prosocial behaviour, higher life satisfaction, better self-rated health, and less problematic conduct [5]. Family emotional expressiveness modulates the child’s emotional understanding, which strongly influences the child’s relationships with peers [6].

Family habits also seem to play a role in child development. Eating habits and behaviour, for example, have a strong influence on a child’s food preferences, diet, and health. This is why Litterbach et al. found that for the child, mealtime with parents is a real opportunity to promote healthy behaviours [7]. Moreover, good family-dinner-related communication, when it also takes into account the wishes and preferences expressed by the child, gives him a sense of control over his meals which makes him more aware, and has proven to be a more effective method than food restrictions in guiding him towards healthier habits [8].

Unfortunately, however, it is often the case that families have no interest in passing on the standards proposed by the WHO regarding children’s physical activity and are even reluctant to enrol children in sports clubs [9]. Experiences are certainly a very important aspect because they allow the child to relate to the world and to grow while learning. However, these are not everything; in fact, it has been shown that the child’s cognitive development has a greater influence than experiences with their family on the level of understanding (concept) the child has about his or her family and its dynamics [10]. One of the most important experiences for a child is play. It contributes to a child’s physical, social, and cognitive development and modulates his or her well-being. It can happen that due to various factors, such as a hurried lifestyle or a change in the family structure, the time set aside for play can be drastically reduced, with serious effects on the child’s development [11].

Family breakdown as a result of divorce represents a traumatic event for a child in itself. This may involve the loss of the known family environment, the need to cope with a forced separation from either parent, and, not infrequently, experiencing parental conflict, which, in some cases, may extend over years, mining the child’s sense of emotional security [12]. Often, children, especially those of pre-school or school age, blame themselves for the dissolution of their parents’ marriage, continuing to fantasise about a family reunion [13,14,15]. Several studies have shown that children of parents who are separated or in the process of divorce exhibit changes in mood, with increased sadness and anger [16,17] and lowered school performance [18,19]. Studies have shown that families in which there is a child diagnosed with autism spectrum disorder (ASD) are more likely to divorce than families of neurotypical children [20]. 

ASD is a neurodevelopmental disorder, of which the specific causes are still unknown, although it is often linked to a damage to neuronal migration in the brain during the prenatal phase [21]. This type of disorder is considered as a set (spectrum) of disorders, as the manifestations vary widely in terms of type and severity. In general, deficits may relate to language, intelligence, social interactions, behaviour, and interests. In the most severe cases, children never learn to speak, and those who do learn to speak do so later than the norm, and echolalia is often present [22]. The conversation rarely occurs interactively and takes place with an unusual vocal rhythm and tone. They do not like to forge interpersonal relationships, and when they relate to other children, they do not use eye contact or facial expressions to establish a social bond [23]. Many people with ASD also have an IQ of less than 70, but have idiosyncratic skills such as performing superior musical skills or performing complex arithmetic calculations in their mind. Happè refers to ASD as a central coherence deficit [24], understood as the ability to combine information in a context to form an overall picture of the situation. Indeed, these individuals have difficulty understanding others’ perceptions of events, their beliefs, motivations, and find it difficult to grasp the context of events. At the behavioural level, they have exaggerated reactions to sensations, and it is frequent that they perform physical and emotional aggression, destruction, and self-injurious actions (beating the head, biting) [25]. These behaviours are named “challenging behaviours” as they are very difficult for parents to handle. Added to those listed above are tantrums, bold and inappropriate statements made to others or in public, verbal or physical perseveration, tuning out, repetitive movements, resistance, and unusual responses [26,27,28]. It has been seen that often when these behaviours appear early on, they can induce hostile reactions and responses in patients, which can lead to the maintenance of the child’s behaviour even years later [29,30].

All these features make ASD a disability with a very high impact on parental well-being [31]. The experience of raising a child with this diagnosis entails a high emotional and financial investment, a high burden of stress, and psychological distress, distancing the parent from good parental satisfaction. The latter is a key indicator of the end of a marriage. 

At the same time, an association between children’s attention-deficit/hyperactivity disorder (ADHD) and parental marital functioning has been demonstrated [32]. 

ADHD is a neurodevelopmental disorder characterised by poor or short attention spans and/or excessive vivacity and impulsivity not appropriate to the child’s age, which interferes with functionality or development [33]. The cause of the disorder is not yet known, although genetic factors are often referred to. Studies indicate that the cause probably lies in alterations at the neurotransmitter level [34,35]. There are certain risk factors such as brain injury or infection, iron deficiency, exposure to lead, alcohol or drugs before birth, and a birth weight of less than 1500 g. A common stereotype is that ADHD results from an overly permissive or non-adaptive parenting style. Leaving aside cases where there is overt neglect or mistreatment, there is very little evidence to support this contention; indeed, it would seem that there are bidirectional and reciprocal effects in place between parent and child [36]. The gravity of symptoms of ADHD may go to mild to severe, and it may be a serious problem in certain environments, particularly in the child’s home or school [37]. Restrictions related to the school environment and an organised lifestyle make attention-deficit/hyperactivity disorder a problem, whereas, in the past, the symptoms may not have significantly interfered with the child’s functions because there was a different understanding of normal child behaviour [38]. The disorder is mainly related to maintaining attention and concentration for the time needed to complete a given task [39]. Sometimes children are impulsive and hyperactive, may forget things easily, be disorganised, and may have difficulties in communication and social interaction [40]. Parents of children who have even a mild variant of the symptoms of this disorder report less marital satisfaction than parents of nonclinical children, increasing the likelihood of divorce [41,42]. In general, it would seem that children with developmental needs, chronic medic conditions, or behavioural problems may cause more stress within the family unit, which would lead to more relationship problems between parents [43]. Therefore, parents tend to respond with unsuccessful attempts to manage such behavioural patterns [44], often including coercion [45], and this increases frustration.

The aim of this review is to evaluate if and how a diagnosis of ASD or ADHD in the child may promote increased divorce within families.

## 2. Materials and Methods

A review of currently published studies was performed following standard guidelines. Online database searches were performed on PubMed and Web of Science for articles published before 1 September 22. It was carried out using the following search keywords and terms (Table 1): (“autism” [All Fields] OR “autisms” [All Fields] OR “autistic disorder” [MeSH Terms] OR (“autistic” [All Fields] AND “disorder” [All Fields]) OR “autistic disorder” [All Fields] OR “autism” [All Fields]) AND (“divorce” [MeSH Terms] OR “divorce” [All Fields] OR “divorced” [All Fields] OR “divorces” [All Fields] OR “divorcing” [All Fields]), AND (“attention deficit disorder with hyperactivity” [MeSH Terms] OR (“attention” [All Fields] AND “deficit” [All Fields] AND “disorder” [All Fields] AND “hyperactivity” [All Fields]) OR “attention deficit disorder with hyperactivity” [All Fields] OR “adhd” [All Fields]) AND (“divorce” [MeSH Terms] OR “divorce” [All Fields] OR “divorced” [All Fields] OR “divorces” [All Fields] OR “divorcing” [All Fields]). 

### 2.1. Inclusion Criteria

**Figure 1:**
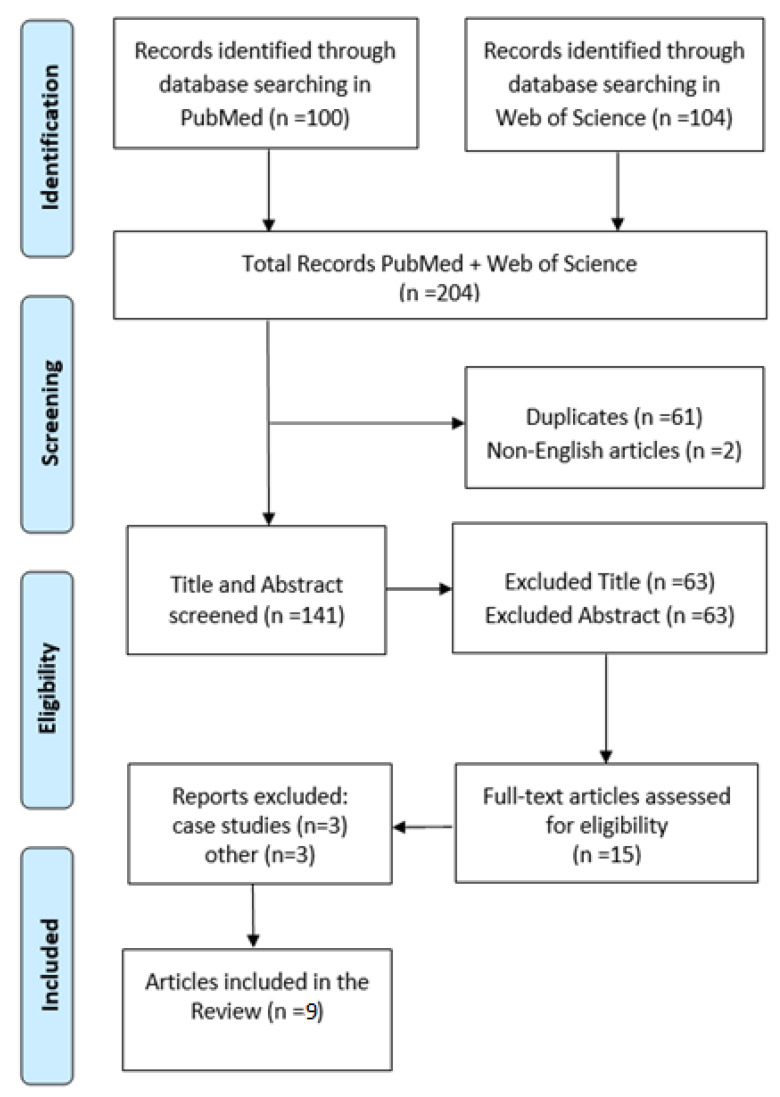
PRISMA flow chart for the study.

A study was included if it examined the concept of divorce in patients diagnosed with ASDor ADHD. Only articles written in English were included in the review. All articles were evaluated based on title and abstract (Figure 1). Studies were included if they were peer-reviewed research, if the sample population included patients with ASD and/or ADHD, and if data compared ASD and ADHD patient performance and health controls (HC) or other diseases.

### 2.2. Exclusion Criteria

Studies were not taken into account if there was a lack of information about divorce, ADHD and/or ASD, if it was a dissertation, commentary, letter, or editorial. No studies were eliminated for this reason. Systematic, integrative, or narrative reviews were also excluded; although, their reference lists were checked and included if appropriate. No restrictions due to the year of publication were adopted.

## 3. Results

### 3.1. ADHD and Divorce

The study by Brown et al. [46] is one of the first to have analysed the correlation between ADHD and family quality of life. In this study, two samples were considered, one of families with children who were diagnosed with ADHD (in the absence of further psychotic disorders or mental retardation IQ > 80) and one in which the child had no type of diagnosis. The aim was to assess the perceptions of parents of children with ADHD regarding their home environment. The children were assessed by psychologists through structured interviews regarding both the symptoms presented and the emotional climate in the home. For parents and teachers, the Conners scales were used. Their results showed that parents of children with ADHD perceived their home environment as less supportive and more stressful than the control sample. Furthermore, it was found that more parents of children with ADHD were physically separated or legally divorced. In another study [47], the authors hypothesised that parents with children with ADHD were more likely to be divorced than parents of neurotypical children. Results of their study show that both parent and child variables likely interact to exacerbate marital discord, and dissolution among families of children diagnosed with ADHD.

Schermerhorn et al. [41], in their paper, examined the mechanisms underlying the association between children’s ADHD and parental marital problems. Their hypothesis was based on the fact that families with children with ADHD had higher rates of separation and divorce than families with children without ADHD. According to the authors, the symptoms of children with ADHD can create more stress for parents by impairing marital functioning. Their study showed that children’s ADHD consistently predicts marital conflict (also controlling for the influence of genetic and environmental factors). In line with this study, Kvist et al. [48] investigated whether having a child with ADHD influences the breakdown of the marital relationship. In this study, a database of 172,299 parent couples was examined, of which 2457 had a first-born child diagnosed with ADHD. Their results over the period considered (1990 to 2007) showed that ten years after the child’s birth, parents of children who had been diagnosed with ADHD were 75% more likely to have divorced. Furthermore, this study took socio-economic status into account, hypothesising that the time spent managing the daily activities of children with ADHD by one or both parents negatively affected their stability, and thus their earnings, reducing and influencing the stability of relationships. 

Finally, Heckel et al. [49] investigated the influence of symptom severity between children with ADHD and parental relationship. A total of 479 children were enrolled. Their results showed that children in the group of separated parents presented significantly more symptoms of inattention, hyperactivity, and impulsivity, and more internalisation/externalisation problems with greater impairment of school and social functioning than children from non-divorced families.

### 3.2. Autism and Divorce

Kalb et al. [50] investigate the separation between parents as a delaying factor in the age at which a child is diagnosed with ASD. Their study was conducted on 561 children receiving a diagnosis of ADS for the first time, and, on average, these children were 5 years old; the evaluation was conducted from 2014 to 2020. The biological parents were asked about their relationship status during the assessment, which categorised them as ‘together’ (married or cohabiting) or ‘not together’ (separated, divorced, or never married). The results showed that children of parents who were together were diagnosed 1.4 years earlier than of those who were not together. This led the authors to highlight the great disparity in age at diagnosis between children whose parents were together during the diagnostic process and those whose were not together, demonstrating the importance of care coordination services for families, particularly when biological parents are not together, identifying the need for systematic supports for single parents.

In contrast, Hartley et al. [20] assessed how impactful the birth of a child with ADS can be on the couple. The authors compared the parents of children with ASD with a sample of parents of children without disabilities. The results showed that the diagnosis of ASD has a negative impact on the couple compared to other disabilities. The main cause is the impairment of social life, brought about by autistic symptoms and associated behaviour, which is often socially ill-tolerated. In a longitudinal study, 391 divorcing parents with adolescents diagnosed with ASD were examined against a sample of 391 parents of children without disabilities. The authors found that up to the age of eight in the children, the difference in separation between parents of children with normal development was on par with parents of children with ADS. Indeed, although different in nature, the stress of raising a child puts a strain on marriages, whether the child has a disability or not. Later on, things change; when children grow up, those without a disability have less need for parental attention and commitment, allowing a renewed focus on the marital relationship, whereas parents of children with an ASD do not experience this condition. This results in couples being exposed to a prolonged period of vulnerability to divorce. Freedman et al. [51] found no evidence that a child with ASD was more likely to live with separated parents. In this study, a nationally representative sample was examined, while Hartley et al. derived information from 391 families of adolescents and adults with ASD in Massachusetts and Wisconsin. In addition, Hartley et al.’s sample examined the relationship of parents of children with ASD to adulthood, which may also have influenced differences in divorce rates, as parents of older children would have had less access to resources and supports that have become more commonly available to families of children with ASD over the past 10–15 years. Moreover, the sample considered by Hartley et al., reflects parents married at a time when divorce rates were higher (1970 and 1980) than those married more recently. Nevertheless, a large body of evidence points to a sharp increase in parenting-related stress, and a decrease in marital satisfaction among parents of children with ASD. Many couples remain together despite predictive indicators of divorce. The reasons could be lack of external social support, emotional, and financial components. According to the authors, the concept of marital stability does not coincide with the concept of marital satisfaction among parents of children with ASD. The National Survey of Children’s Health (NSCH) used a telephone survey to identify households within which there were children under the age of 18. Between 2007 and 2008, the NSCH surveyed a total of 91,642 parents of children between the ages of 3 and 17. The results showed that there is no correlation between a child with ASD and living in a two-parent family, compared to living in a family with separated parents. However, children with externalising, internalising, and ADHD disorders, regardless of whether or not they have ASD, are less likely to live in a two-parent household. One possible explanation for this result could be that families with a child with ASD are more likely to receive support services for their disability (e.g., through school programs) than children without ASD who have psychiatric disorders, whose abnormal behaviour, in measure also of its severity, e.g., aggression and hyperactivity, is even more pronounced at marital dissolution, even more so than intellectual retardation. As for parents of children with intellectual disability, they have to face many challenges on a daily basis due to cognitive, motor, medical or psychopathological deficits, which usually give rise to strong emotional reactions and worries. The child can usually look different than his peers and exhibit different behaviours. The parent has to manage all aspects of the child’s life and worries begin early for the adult about the child’s future. The presence of a child with intellectual disability can cause marital difficulties in the parental couple: mothers often become overprotective of their child and focus all their attention on the child, while fathers tend to distance themselves from the problem, both physically and mentally. Separation and divorce rates are frequent in cases of parents of children with mental retardation [52].

## 4. Discussions

As seen, the severity of symptoms of children affected by different forms of psychopathology has been associated with parental stress in various studies. It seems that both the typical and characteristic symptoms of the disorder, and the difficulties in communication or in parent relationships affect the level of stress perceived by the parents. 

From the literature concerning the parents of children with ASD, it has emerged that stress occupies a time frame that affects both the periods before and after diagnosis. Finally, after a long search, the child receives the diagnosis of ASD and very often high levels of stress are reported, even if not all parents of children with this disorder report them. In 2011, Johnson et al. conducted a study regarding parental stress, functioning, and health-related quality of life in families with children and adolescents affected by ASD [53]. They found that parents reported a high level of stress, also given by the decisions they had to make on a daily basis, in managing their child and his problems. Stress could also be caused by different factors, including the quality of social support and relationships, the quality of family functioning, and the expectations held by the parents. In another study [54], the stress of mothers and fathers of younger children with ASD was analysed, and also, in this study, parents reported high levels of stress. Problems in the children’s social and relational skills were associated with stress and problems in interacting with the child. Maternal stress was associated with infant regulation difficulties, while father stress was more associated with behavioural problems. 

Parents of children with pervasive developmental disorders (PDD) usually have higher levels of parental stress compared to other parents with typically developing children, but also compared to parents of children with other disabilities. Various studies have investigated parental stress in families with children with PDD, and what emerged was that families of children with ASD were more stressed than families without children with these disorders [55]. Child behaviour problems are associated with high levels of parental stress, more than developmental delay. For example, Lecavalier et al. showed that specific behaviour problems were more predictive of high stress than adaptive behaviours [56]. It would seem that the needs of the families of children with ASD are more specific than usual [57], and most frequent complaints from parents of children with disabilities are related to the communication and motor difficulties of their children [58]. 

As shown by Beck et al. [59], even positive factors can influence stress; in fact, they found that both limited prosocial behaviours and behavioural problems were associated with high levels of stress. Parents of children with ASD score higher on levels of stress than other groups of parents [60]. The daily challenges of caring for the child are endless, and affect all aspects of the child’s care as well as the parent’s mental health and ability to manage the needs of the child and family [61]. In the beginning, when a diagnosis has not yet been made, parents strenuously try to understand their child’s behavioural and communication problems [62,63,64,65], and when they are unable to give themselves an answer, they turn to different specialists [66,67,68,69,70,71], sometimes taking a long time. After the diagnosis, caregivers and family members of children with ASD experience feelings of sadness, guilt, and mourning for the discovery of the impossibility of curing the syndrome, revealing the need for care for this family [71,72,73,74]. These parents seem to have a characteristic profile; indeed, Koegel et al. [75] found specific areas of difficulty for parents raising their children with ASD. The differences that they found in their study with parents of typically developing children were concerns about the child’s future, cognitive ability and autonomy, and integration into the community [76,77,78]. Similarly, Holroyd and MacArthur [79] made a comparison between mothers of children with ASD and mothers of children with Down Syndrome (DS). They found that mothers of children with ASD reported that their children were less autonomous and more physically dependent. The most important influential factors of these families’ qualities of life were whether the child with ASD had a major health concern and if the family’s needs were met by disability-related services, and whether there were opportunities to engage in recreational activities [80]. Furthermore, it would appear that parental stress is influenced by specific stressful issues, such as disadvantages due to the situation. In the aforementioned study by Koegel et al. [75], the authors reported that the opportunities of these families were more limited than those of families with parents of healthy children, and that these parents are more inclined to take care of the child within the home rather than spend time engaging in leisure activities outside the home. In parents, future aspirations and dreams, relationships with friends and family, and daily routines change profoundly [81].

The level of stress experienced by mothers and fathers has also been compared and the results are not unequivocal. In some respects, it would seem that mothers experience on average more stress than fathers [82]. These results could be seen as a natural consequence of the more central role played by the mother in the care of the child with ASD. However, more recent studies state that the level of stress experienced by mothers and fathers is the same. This could be seen as a consequence of the fact that fathers over time have increasingly taken on more responsibility for their children, with this disorder leading to a more equal distribution of tasks [83]. Family emotional climate in having a child with ASD was assessed in a study by Hickey et al. [84], which found that 43% of these families were characterised by high levels of warmth and low levels of criticism both in parent–child and parent–couple relationships. Moreover, Feldman et al. reported that parents engaging in accommodation at least once a month and family accommodation was significantly positively correlated with restricted and repetitive behaviour severity [85]. In parents of children with ASD, it seems that even the choice of the stress management strategies can change the family mood. Adopting problem-solving strategies, seeking social support, implementing positive reframing, implementing emotional regulation, or a negotiation or compromise-seeking strategy is associated with an increase in mood. On the other hand, an avoidance strategy of the stressful situation, attempt to distract oneself, a distancing strategy from others, and a resignation response led to a decrease in positive mood [86]. Finally, it should be mentioned that some studies have shown that families’ perceived stress tends to decrease over time as a result of family adjustment [87], understanding, and acceptance of their condition [88], and the development of a more regular routine [89].

With regard to children with ADHD, often their poor school performance and lack of commitment shown in doing homework is interpreted by adults as oppositional behaviour, or as a sign of lack of responsibility or laziness. However, positive parenting styles seem to protect children with ADHD [90]; in fact, high affective warmth and strong boundaries have been shown to improve social competence in children with ADHD [91]. Unfortunately, within their families, there are often conflicts, resentments, and antagonism [92]: the variety of symptoms exhibited by children might make parents think there is intentionality behind his actions. Often the mother of a child with ADHD makes the choice to leave her professional activity to devote herself completely to managing the child and the home. The parent must face the fact that the child needs a great deal of time compared to other children; the adult must often be present and guide the child in the various daily activities [93]. The performance of tasks that might seem as simple as getting the children go to sleep, getting them ready for school, feeding, or doing homework can be problematic. Frustration is frequent in the parent; in fact, it seems to establish a vicious circle of conflicts within the couple, and educational inconsistencies are often created [48]. The child’s behaviours are often perceived as annoying and stressful and arouse strong reactions in the parent, who imparts more severe forms of punishment. In general, parents of children with ADHD show less confidence in their parenting knowledge, compared with parents of neurotypical children [94]. Mothers of children with ADHD have less positive, more stressful, and unfulfilling interactions with their children and even social isolation, self-blame, and depression than mothers of typically developing children [95].

For these parents, having a child with these problems can be a source of stress, low self-esteem, and can change the parent’s perception of their parental role, the characteristics of the child, and the type of parent–child interaction [94]. As demonstrated in the National Survey of Children’s Health study, children with externalising, internalising, and ADHD disorders, regardless of whether or not they have ASD, are less likely to live in a two-parent household. This is probably due to the fact that families with a child with ASD are more likely to receive support services for their disability (e.g., through school programs) than children without ASD who have psychiatric disorders, whose abnormal behaviour, in measure also of its severity is even more pronounced at marital dissolution. However, while wanting to compare the two conditions, it is not yet possible to determine in which of the two conditions the impact on the family is worse, or whether it is equal. What we do know is that in both cases, the impact on the couple’s relationship is highly destabilising, since, unlike other pathological conditions, they can lead to physical and emotional stress, which is incredibly demanding; moreover, the constant presence to which the partners are called upon can lead to work impairment and a consequent decrease in the family’s financial resources. It can be inferred from these studies that the management of children with ASD or ADHD takes a heavy toll on marriages, while others have a minimal impact. The high risk of divorce in parents of children with an ASD is therefore similar to the high divorce rate found in parents of children with ADHD; although, in the latter, it would appear to be slightly higher, probably due to the fact that in families in which there is a child with an ASD, as well as benefiting from family support even earlier, the parents have more time to adjust to a more appropriate relationship with their child, as the condition is more evident from the earliest years of life, while ADHD is more likely to be diagnosed later; however, they are still predictors of a higher risk of divorce than parents of children with normal development. Parental potential is also determined, as some may have more limited resources to cope with these demands, which may increase their risk of family breakdown, including marital dissolution. One of the limitations of this study could be identified in not being able to give an account of the real effect that ASD and ADHD have in separation or divorce. The increased likelihood that couples have of divorce could in fact also be due to poor coping skills in parents, lack of tolerance, feeling of unpreparedness, the little help received, and lack of services. Moreover, the scientific literature could use more rigorous methods and standardized tools. 

It is often assumed that the child’s well-being controls the stress level of the family and parents (i.e., if the child is well, the family is less stressed); research, however, is beginning to emphasise the importance of parental and family serenity and its direct implications on child well-being. All of this suggests that the ability of parents to maintain a positive outlook can have an impact on their parental stress, implying a greater complicity beneficial to the child’s growth, thus reinforcing the importance of care coordination/family support services offered to families, with support services being able to serve as a mediating variable to prevent divorce or separation of couples. Thus, finding coping strategies to teach parents, and guiding them in identifying proactive attitudes to improve their marital relationship could be an idea for support interventions to strengthen the family system, and a support for parents, which could be a significant predictor of marital stability. It would also be desirable for such support not to stop in the early years of the child’s life, but to continue over the years, as parents of children with such disabilities continue to experience marital strain into adulthood, facing a unique set of challenges as their child grows older, including assisting in the transition from school to work, in the community, and in long-term care planning, which can add new strains to the parents’ marriage.

## Figures and Tables

**Table 1:** Table of the terms used in the query in both databases.

Database	Query Terms
PubMed	“Autism” OR “autisms” OR “autistic disorder” OR “autistic” AND (“disorder” OR “autistic disorder” OR “autism”) AND (“divorce” OR “divorce” OR “divorced” OR “divorces” OR “divorcing”), AND (“attention deficit disorder with hyperactivity” OR (“attention” AND “deficit” AND “disorder” AND “hyperactivity”) OR (“attention deficit disorder with hyperactivity” OR “adhd”) AND (“divorce” OR “divorce” OR “divorced” OR “divorces” OR “divorcing”).
Web of Science	

## Data Availability

Not applicable.

## References

[1] Tabachnick A.R., He Y., Zajac L., Carlson E.A., Dozier M. (2022). Secure attachment in infancy predicts context-dependent emotion expression in middle childhood. Emotion.

[2] Gewirtz A.H., Zamir O. (2014). The impact of parental deployment to war on children: The crucial role of parenting. Adv. Child Dev. Behav..

[3] Posada G., Lü T., Trumbell J., Kaloustian G., Trudel M., Plata S.J., Peña P.P., Perez J., Tereno S., Dugravier R. (2013). Is the Secure Base Phenomenon Evident Here, There, and Anywhere? A Cross-Cultural Study of Child Behavior and Experts’ Definitions. Child Dev..

[4] Hammes P.S., Crepaldi M.A., Bigras M. (2012). Family Functioning and Socioaffective Competencies of Children in the Beginning of Schooling. Span. J. Psychol..

[5] Herke M., Knöchelmann A., Richter M. (2020). Health and Well-Being of Adolescents in Different Family Structures in Germany and the Importance of Family Climate. Int. J. Environ. Res. Public Health.

[6] Cassidy J., Parke R.D., Butkovsky L., Braungart J.M. (1992). Family-Peer Connections: The Roles of Emotional Expressiveness within the Family and Children’s Understanding of Emotions. Child Dev..

[7] Litterbach E.-K.V., Campbell K.J., Spence A.C. (2017). Family meals with young children: An online study of family mealtime characteristics, among Australian families with children aged six months to six years. BMC Public Health.

[8] Alm S., Olsen S.O., Honkanen P. (2015). The role of family communication and parents’ feeding practices in children’s food preferences. Appetite.

[9] Rakha A.H., Albahadel D.M., Saleh H.A. (2022). Developing an active lifestyle for children considering the Saudi vision 2030: The family’s point of view. PLoS ONE.

[10] Mann B.J., Borduin C.M., Cone L.T., Borduin B.J., Sylvester C.E. (1992). Children’s Concepts of the Family. J. Am. Acad. Child Adolesc. Psychiatry.

[11] Ginsburg K.R., American Academy of Pediatrics Committee on Communications, American Academy of Pediatrics Committee on Psychosocial Aspects of Child and Family Health (2007). The Importance of Play in Promoting Healthy Child Development and Maintaining Strong Parent-Child Bonds. Pediatrics.

[12] Davies P.T., Cummings E.M. (1994). Marital conflict and child adjustment: An emotional security hypothesis. Psychol. Bull..

[13] Kleinsorge C., Covitz L.M. (2012). Impact of Divorce on Children: Developmental Considerations. Pediatr. Rev..

[14] Hirsch M. (2001). Schuld und Schuldgefühl im Zusammenhang mit Trennung und Scheidung [Guilt and subjective feelings of guilt in the context of separation and divorce]. Prax. Der Kinderpsychol. Und Kinderpsychiatr..

[15] van Dijk R., van der Valk I.E., Deković M., Branje S. (2022). Triangulation and child adjustment after parental divorce: Underlying mechanisms and risk factors. J. Fam. Psychol..

[16] Spremo M. (2020). Children and Divorce. Psychiatr. Danub..

[17] Leung A.K., Robson L.M. (1990). Children of Divorce. J. R. Soc. Health.

[18] Çaksen H. (2021). The effects of parental divorce on children. Psychiatriki.

[19] Nunes-Costa R.A., Lamela D.J.P.V., Figueiredo B.F.C. (2009). Psychosocial adjustment and physical health in children of divorce. J. Pediatr..

[20] Hartley S.L., Barker E.T., Seltzer M.M., Floyd F., Greenberg J., Orsmond G., Bolt D. (2010). The relative risk and timing of divorce in families of children with an autism spectrum disorder. J. Fam. Psychol..

[21] Watts T.J. (2008). The Pathogenesis of Autism. Clin. Pathol..

[22] Lord C., Elsabbagh M., Baird G., Veenstra-Vanderweele J. (2018). Autism spectrum disorder. Lancet.

[23] Kodak T., Bergmann S. (2020). Autism Spectrum Disorder. Pediatr. Clin. N. Am..

[24] Happé F., Frith U. (2006). The Weak Coherence Account: Detail-focused Cognitive Style in Autism Spectrum Disorders. J. Autism Dev. Disord..

[25] Fodstad J.C., Rojahn J., Matson J.L. (2012). The Emergence of Challenging Behaviors in At-Risk Toddlers with and without Autism Spectrum Disorder: A Cross-Sectional Study. J. Dev. Phys. Disabil..

[26] Ludlow A., Skelly C., Rohleder P. (2011). Challenges faced by parents of children diagnosed with autism spectrum disorder. J. Health Psychol..

[27] Bearss K., Johnson C., Handen B., Smith T., Scahill L. (2012). A Pilot Study of Parent Training in Young Children with Autism Spectrum Disorders and Disruptive Behavior. J. Autism Dev. Disord..

[28] Myers B.J., Mackintosh V.H., Goin-Kochel R.P. (2009). My greatest joy and my greatest heartache: Parents’ own words on how having a child in the autism spectrum has affected their lives and their families’ lives. Res. Autism Spectr. Disord..

[29] Harold G.T., Leve L.D., Barrett D., Elam K., Neiderhiser J.M., Natsuaki M.N., Shaw D.S., Reiss D., Thapar A. (2013). Biological and rearing mother influences on child ADHD symptoms: Revisiting the developmental interface between nature and nurture. J. Child Psychol. Psychiatry.

[30] Harold G.T., Leve L.D., Elam K.K., Thapar A., Neiderhiser J.M., Natsuaki M.N., Shaw D.S., Reiss D. (2013). The nature of nurture: Disentangling passive genotype–environment correlation from family relationship influences on children’s externalizing problems. J. Fam. Psychol..

[31] Masi A., DeMayo M.M., Glozier N., Guastella A.J. (2017). An Overview of Autism Spectrum Disorder, Heterogeneity and Treatment Options. Neurosci. Bull..

[32] Johnston C., Mash E.J. (2001). Families of Children With Attention-Deficit/Hyperactivity Disorder: Review and Recommendations for Future Research. Clin. Child Fam. Psychol. Rev..

[33] Cortese S., Coghill D. (2018). Twenty years of research on attention-deficit/hyperactivity disorder (ADHD): Looking back, looking forward. Evid. Based Ment. Health.

[34] Sharma A., Couture J. (2013). A Review of the Pathophysiology, Etiology, and Treatment of Attention-Deficit Hyperactivity Disorder (ADHD). Ann. Pharmacother..

[35] Tripp G., Wickens J.R. (2009). Neurobiology of ADHD. Neuropharmacology.

[36] Bell R.Q. (1968). A reinterpretation of the direction of effects in studies of socialization. Psychol. Rev..

[37] Hechtman L. (1996). Families of Children with Attention Deficit Hyperactivity Disorder: A Review. Can. J. Psychiatry.

[38] Hinshaw S.P. (2018). Attention Deficit Hyperactivity Disorder (ADHD): Controversy, Developmental Mechanisms, and Multiple Levels of Analysis. Annu. Rev. Clin. Psychol..

[39] Coxe S., Sibley M.H., Becker S.P. (2020). Presenting problem profiles for adolescents with ADHD: Differences by sex, age, race, and family adversity. Child Adolesc. Ment. Health.

[40] Posner J., Polanczyk G.V., Sonuga-Barke E. (2020). Attention-deficit hyperactivity disorder. Lancet.

[41] Schermerhorn A.C., D’Onofrio B.M., Slutske W.S., Emery R.E., Turkheimer E., Harden K.P., Heath A.C., Martin N.G. (2012). Offspring ADHD as a Risk Factor for Parental Marital Problems: Controls for Genetic and Environmental Confounds. Twin Res. Hum. Genet..

[42] Mohammadi M.R., Farokhzadi F., Alipour A., Rostami R., Dehestani M., Salmanian M. (2012). Marital Satisfaction amongst Parents of Children with Attention Deficit Hyperactivity Disorder and Normal Children. Iran. J. Psychiatry.

[43] Neece C.L., Green S.A., Baker B.L. (2012). Parenting Stress and Child Behavior Problems: A Transactional Relationship Across Time. Am. J. Intellect. Dev. Disabil..

[44] Beauchaine T.P., McNulty T. (2013). Comorbidities and continuities as ontogenic processes: Toward a developmental spectrum model of externalizing psychopathology. Dev. Psychopathol..

[45] Patterson G.R. (1982). Coercive Family Process.

[46] Brown R.T., Pacini J.N. (1989). Perceived family functioning, marital status, and depression in parents of boys with at-tention deficit disorder. J. Learn. Disabil..

[47] Wymbs B.T., Pelham W.E., Molina B.S., Gnagy E.M., Wilson T.K., Greenhouse J.B. (2008). Rate and predictors of divorce among parents of youths with ADHD. J. Consult. Clin. Psychol..

[48] Kvist A.P., Nielsen H.S., Simonsen M. (2013). The importance of children’s ADHD for parents’ relationship stability and labor supply. Soc. Sci. Med..

[49] Heckel L., Clarke A., Barry R., McCarthy R., Selikowitz M. (2009). The relationship between divorce and the psychological well-being of children with ADHD: Differences in age, gender, and subtype. Emot. Behav. Difficulties.

[50] Kalb L.G., Holingue C., Pfeiffer D., Reetzke R., Dillon E., Azad G., Freedman B., Landa R. (2021). Parental relationship status and age at autism spectrum disorder diagnosis of their child. Autism.

[51] Freedman B.H., Kalb L.G., Zablotsky B., Stuart E. (2011). Relationship Status Among Parents of Children with Autism Spectrum Disorders: A Population-Based Study. J. Autism Dev. Disord..

[52] Bornstein M.H. (2002). Parenting Infants. Handbook of Parenting, Volume 1: Children and Parenting.

[53] Johnson N., Frenn M., Feetham S., Simpson P. (2011). Autism spectrum disorder: Parenting stress, family functioning and health-related quality of life. Fam. Syst. Health.

[54] Davis N.O., Carter A.S. (2008). Parenting Stress in Mothers and Fathers of Toddlers with Autism Spectrum Disorders: Associations with Child Characteristics. J. Autism Dev. Disord..

[55] Lecavalier L., Leone S., Wiltz J. (2006). The impact of behaviour problems on caregiver stress in young people with autism spectrum disorders. J. Intellect. Disabil. Res..

[56] Losada-Puente L., Baña M., Asorey M.J.F. (2022). Family quality of life and autism spectrum disorder: Comparative diagnosis of needs and impact on family life. Res. Dev. Disabil..

[57] Bertule D., Vetra A. (2014). The family needs of parents of preschool children with cerebral palsy: The impact of child’s gross motor and communications functions. Medicina.

[58] Beck A., Hastings R.P., Daley D., Stevenson J. (2004). Pro-social behaviour and behaviour problems independently predict maternal stress. J. Intellect. Dev. Disabil..

[59] Bonis S. (2016). Stress and Parents of Children with Autism: A Review of Literature. Issues Ment. Health Nurs..

[60] Magalhães J.M., Rodrigues T.A., Neta M.M.R., Damasceno C.K.C.S., Sousa K.H.J.F., Arisawa E.L.S. (2021). Experiences of family members of children diagnosed with autism spectrum disorder. Rev. Gaúcha Enferm..

[61] Meadan H., Halle J.W., Ebata A.T. (2010). Families with Children Who Have Autism Spectrum Disorders: Stress and Support. Except. Child..

[62] Duarte C.S., Bordin I.A., Yazigi L., Mooney J. (2005). Factors associated with stress in mothers of children with autism. Autism.

[63] Faraone S.V., Asherson P., Banaschewski T., Biederman J., Buitelaar J.K., Ramos-Quiroga J.A., Rohde L.A., Sonuga-Barke E.J., Tannock R., Franke B. Attention-deficit/hyperactivity disorder. https://www.nature.com/articles/nrdp201520.

[64] Montes G., Halterman J.S. (2007). Psychological functioning and coping among mothers of children with autism: A pop-ulation-based study. Pediatrics.

[65] Silva LM T., Schalock M. (2012). Autism Parenting Stress Index: Initial psychometric evidence. J. Autism Dev. Disord..

[66] Braddock B., Twyman K. (2014). Access to Treatment for Toddlers with Autism Spectrum Disorders. Clin. Pediatr..

[67] Dabrowska A., Pisula E. (2010). Parenting stress and coping styles in mothers and fathers of pre-school children with autism and Down syndrome. J. Intellect. Disabil. Res..

[68] Mooney E.L., Gray K.M., Tonge B.J. (2006). Early features of autism: Repetitive behaviors in young children. Eur. Child Adolesc. Psychiatry.

[69] Moh T.A., Magiati I. (2012). Factors associated with parental stress and satisfaction during the process of diagnosis of children with Autism Spectrum Disorders. Res. Autism Spectr. Disord..

[70] Altiere M.J., von Kluge S. (2009). Searching for acceptance: Challenges encountered while raising a child with autism. J. Intellect. Dev. Disabil..

[71] Nealy C.E., O’Hare L., Powers J.D., Swick D.C. (2012). The Impact of Autism Spectrum Disorders on the Family: A Qualitative Study of Mothers’ Perspectives. J. Fam. Soc. Work..

[72] Lutz H.R., Patterson B.J., Klein J. (2012). Coping with Autism: A Journey Toward Adaptation. J. Pediatr. Nurs..

[73] Neely-Barnes S.L., Hall H.R., Roberts R.J., Graff J.C. (2011). Parenting a Child with an Autism Spectrum Disorder: Public Perceptions and Parental Conceptualizations. J. Fam. Soc. Work..

[74] Dunn M.E., Burbine T., Bowers C.A., Tantleff-Dunn S. (2001). Moderators of Stress in Parents of Children with Autism. Community Ment. Health J..

[75] Koegel L.K., Koegel R.L., Hurley C., Frea W.D. (1992). Improving social skills and disruptive behavior in children with autism through self-management. J. Appl. Behav. Anal..

[76] Jones L., Totsika V., Hastings R.P., Petalas M.A. (2013). Gender differences when parenting children with autism spec-trum disorders: A multilevel modeling approach. J. Autism Dev. Disord..

[77] Hill-Chapman C.R., Herzog T.K., Maduro R.S. (2013). Aligning over the child: Parenting alliance mediates the association of autism spectrum disorder atypicality with parenting stress. Res. Dev. Disabil..

[78] Ingersoll B., Hambrick D.Z. (2011). The relationship between the broader autism phenotype, child severity, and stress and depression in parents of children with autism spectrum disorders. Res. Autism Spectr. Disord..

[79] Holroyd J., McArthur D. (1976). Mental retardation and stress on the parents: A contrast between Down’s syndrome and childhood autism. Am. J. Ment. Defic..

[80] Jones S., Bremer E., Lloyd M. (2016). Autism spectrum disorder: Family quality of life while waiting for intervention services. Qual. Life Res..

[81] Dale E., Jahoda A., Knott F. (2006). Mothers’ attributions following their child’s diagnosis of autistic spectrum disorder: Exploring links with maternal levels of stress, depression, and expectations about their child’s future. Autism.

[82] Herring S., Gray K., Taffe J., Tonge B., Sweeney D., Einfeld S. (2006). Behaviour and emotional problems in toddlers with pervasive developmental disorders and developmental delay: Associations with parental mental health and family functioning. J. Intellect. Disabil. Res..

[83] Hastings R.P., Kovshoff H., Brown T., Ward N.J., Degli Espinosa F., Remington B. (2005). Coping strategies in mothers and fathers of preschool and school-age children with autism. Autism.

[84] Hickey E.J., Nix R.L., Hartley S.L. (2019). Family Emotional Climate and Children with Autism Spectrum Disorder. J. Autism Dev. Disord..

[85] Feldman I., Koller J., Lebowitz E.R., Shulman C., Ben Itzchak E., Zachor D.A. (2019). Family Accommodation in Autism Spectrum Disorder. J. Autism Dev. Disord..

[86] Pottie C.G., Cohen J., Ingram K.M. (2008). Parenting a Child with Autism: Contextual Factors Associated with Enhanced Daily Parental Mood. J. Pediatr. Psychol..

[87] Bekhet A.K., Johnson N.L., Zauszniewski J.A. (2012). Resilience in Family Members of Persons with Autism Spectrum Disorder: A Review of the Literature. Issues Ment. Health Nurs..

[88] Rautakoski P., Ursin P.A., Carter A.S., Kaljonen A., Nylund A., Pihlaja P. Communication Skills Predict Social-Emotional Competencies. https://www.sciencedirect.com/science/article/pii/S0021992421000617.

[89] Gray D.E. (2006). Coping over time: The parents of children with autism. J. Intellect. Disabil. Res..

[90] Healey D.M., Flory J.D., Miller C.J., Halperin J.M. (2011). Maternal positive parenting style is associated with better functioning in hyperactive/inattentive preschool children. Infant Child Dev..

[91] Hinshaw S.P., Zupan B.A., Simmel C., Nigg J.T., Melnick S.M. (1997). Peer status in boys with and without attention-deficit hyper-activity disorder: Predictions from overt and covert antisocial behavior, social isolation, and authoritative parenting beliefs. Child Dev..

[92] Hurtig T., Ebeling H., Taanila A., Miettunen J., Smalley S., McGough J., Loo S., Järvelin M.-R., Moilanen I. (2007). ADHD and comorbid disorders in relation to family environment and symptom severity. Eur. Child Adolesc. Psychiatry.

[93] Deater-Deckard K. (2017). Parents’ and Children’s ADHD in a Family System. J. Abnorm. Child Psychol..

[94] Mash E.J., Johnston C. (1983). Parental perceptions of child behavior problems, parenting self-esteem, and mothers’ reported stress in younger and older hyperactive and normal children. J. Consult. Clin. Psychol..

[95] Barkley R.A. (1997). Behavioral inhibition, sustained attention, and executive functions: Constructing a unifying theory of ADHD. Psychol. Bull..

